# *Staphylococcus epidermidis* in Acute Myeloid Leukemia: A Comparative Genomic Study Against Non-AML Isolates

**DOI:** 10.3390/pathogens14070627

**Published:** 2025-06-24

**Authors:** Stephanie McMahon, Samantha Franklin, Maliha Batool, Nitya Sadasivan, Safa Fatima, Jessica Galloway-Peña

**Affiliations:** Laboratory of Jessica Galloway-Peña, Interdisciplinary Genetics Program, Department of Veterinary Pathobiology, Texas A&M University, College Station, TX 77843, USA; stephmcm@tamu.edu (S.M.); srfranklin@tamu.edu (S.F.); mbatool@cvm.tamu.edu (M.B.); nsadasivan@tamu.edu (N.S.); safachamp@tamu.edu (S.F.)

**Keywords:** acute myeloid leukemia, comparative genomics, phylogeny, antibiotic resistance, virulence, *Staphylococcus epidermidis*

## Abstract

Bloodstream infections (BSIs) are a major cause of morbidity and mortality in acute myeloid leukemia (AML) patients undergoing induction chemotherapy. *Staphylococcus epidermidis*, typically a skin commensal, is increasingly recognized as a pathogen in these vulnerable individuals. This study investigated whether genomic differences exist between infectious and gastrointestinal colonizing *S. epidermidis* isolates from AML patients and how these compare to colonizing and infectious isolates from other patient groups and biogeographic sites. We analyzed 114 isolates—44 from AML patients (23 infections, 21 GI colonizers) and 70 from public datasets (34 infections, 36 colonizers). Stool samples underwent 16S rRNA sequencing and culture to identify colonization, while bloodstream isolates were sequenced and compared. Genomic profiling using Roary, Scoary, Phyre2, and InterProScan revealed that infectious and GI-colonizing AML isolates were phylogenetically close but genomically distinct. Infectious isolates from AML patients were significantly enriched for resistance genes (e.g., *mecA*, *mecR1*, *mecI*, *ANT(4′)-Ib*) and the biofilm-associated gene *icaA*. AML infectious isolates harbored more resistance genes and mobile elements than non-AML strains but lacked widespread classical virulence factors. These results suggest that *S. epidermidis* pathogenicity in immunocompromised hosts is driven by genomic adaptability and antibiotic tolerance rather than traditional virulence mechanisms.

## 1. Introduction

Bloodstream infections (BSIs) pose a significant risk for morbidity and mortality among immunocompromised patients, but specifically among acute myeloid leukemia (AML) patients undergoing chemotherapy [1,2,3]. AML is the most common form of acute leukemia affecting adults, and patients are commonly treated with extensive regimens of cytotoxic chemotherapy [4]. Due to the nature of the disease, AML patients suffer from severely compromised immune systems, further exacerbated by treatment with chemotherapy and antibiotic treatment [5,6].

IDSA guidelines recommend universal prophylaxis, as well as broad-spectrum empirical antibiotic administration, for all patients experiencing fever in the setting of neutropenia, which is ~80% of all AML patients undergoing induction chemotherapy [7]. Despite these increased instances of neutropenic fever, only 20% to 30% of febrile cases result in clinically documented infection [3,7]. However, the prophylactic and empirical antibiotic treatment not only disrupts the symbiotic relationship between the host and the gut microbiome, but it also disturbs the complex bacterial networks that exist in the microbiome while exacerbating intestinal damage to the mucosal layer caused by intensive cytotoxic chemotherapy [8,9]. Consequently, this combination of increased antibiotic administration and intense chemotherapy leaves patients highly susceptible to infection via translocation from the gastrointestinal tract [10,11,12]. Common pathogens implicated in such translocations include Gram-negative bacilli like *Escherichia coli*, *Klebsiella pneumoniae*, and *Pseudomonas aeruginosa*, as well as Gram-positive organisms such as *Enterococcus faecalis* and *Streptococcus* species [13,14]. These bacteria, while part of the normal gut flora, can cause bloodstream infections when the mucosal barrier is compromised.

While *Staphylococcus epidermidis* has been shown to account for approximately 20% of bloodstream infections in AML patients and is increasingly recognized as an opportunistic pathogen in this context, there are a lack of studies examining the epidemiology of gastrointestinal colonization by *S. epidermidis* in immunocompromised patient populations [4,15,16]. This gap in knowledge underscores the need to understand the phylogeny of *S. epidermidis* colonizing the gastrointestinal tract and those strains’ potential contribution to transmission or systemic infections in vulnerable patient cohorts.

*Staphylococcus epidermidis* is a ubiquitous skin commensal that normally exists harmlessly as part of the human microflora [17,18]. However, it has recently emerged as a significant opportunistic pathogen, particularly in immunocompromised patients, where it accounts for a substantial proportion of bloodstream infections (BSIs) [19]. Notably, specific multi-locus sequence types (STs) such as ST2 and ST5 are frequently associated with invasive infection, including BSIs, and are characterized by multidrug resistance and enhanced virulence factors like genes encoding biofilm formation capabilities [20]. While less common than ST2 and ST5, ST54 has also been identified among BSI cases in healthcare settings [21]. In contrast, the most frequently reported sequence types among colonization isolates is ST2 ST59, ST22, and ST5 [22,23].

The pathogenic potential of *S. epidermidis* is further impacted by the presence or absence of various antimicrobial resistance genes (ARGs) and virulence factors. Genes such as *mecA*, conferring methicillin resistance, and *icaADBC*, associated with biofilm production, are prevalent among invasive strains [24]. Additionally, elements like arginine catabolic mobile element (ACME) and phenol-soluble modulins (PSMs) contribute to the bacterium’s ability to evade the host immune response and establish persistent infection [25]. Understanding the genetic distinction between colonizing and infectious isolates, particularly in the gastrointestinal tract, is crucial for elucidating the mechanisms of transmission and infection and the potential for translocation in vulnerable patient cohorts. In this manuscript, we sought to understand how phylogenetics, compositions of antibiotic resistance and virulence genes, and the pan-genome differed between *S. epidermidis* isolates stemming from different patient populations and different sources.

## 2. Materials and Methods

### 2.1. Stool Sample Collection, 16s rRNA Sequencing, and Determination of GI-Colonizing Isolates

Stool samples were collected from three distinct AML patient groups undergoing remission induction chemotherapy (IC) at MD Anderson Cancer Center in Houston, Texas. The first group (PA14-0641) consisted of 63 AML patients part of a previously published study who were enrolled from September 2014 to January 2019, where infectious isolates were collected at the time of positive blood culture [4]. The second group, enrolled between September 2013 and August 2015 (PA13-0339), included 98 AML patients. Stool samples were taken every 96 hours during IC when available, ceasing once neutrophil recovery occurred, as previously described [26]. The third group (PA15-0780), which included AML patients undergoing IC enrolled from January 2015 to February 2020, had stool samples collected biweekly during the first 4 weeks, weekly from weeks 4 to 8, every two weeks from weeks 8 to 12, and then every other week up to 24 weeks, or until loss to follow-up. All infectious/bloodstream isolates collected by the clinical microbiology laboratory while patients were on study for PA13-0339 and PA15-0780 were obtained as described previously [26].

Genomic DNA was extracted from stool samples by utilizing the QIAamp Fast DNA Stool Mini Kit (Qiagen, Hilden, Germany), with modification to the established protocol to include an additional bead-beating lysis step [27]. The V4 region of the 16S rRNA gene was amplified using Illumina Miseq (Illumina, San Diego, CA, USA) using a 2 × 250 bp paired-end protocol [26,28,29]. The sequences were combined, dereplicated, and filtered for length using VSEARCH. After denoising and identifying chimeras with UNOISE3 commands, the distinct sequences, or zero-radius zOTUs, were assigned taxonomic classifications using Mothur (version 1.43.0) in conjunction with the SILVA database version 138. Alpha and beta diversity indices were calculated using QIIME 2 [30,31]. The 16S rRNA sequences from PA13 stool samples have been previously published and are available in the NCBI Sequence Read Archive under Bioproject IDs PRJNA352060 and PRJNA526551 [26,28,29]. The 16S rRNA sequences for the PA15 cohort are also archived in the NCBI Sequence Read Archive under Bioproject number PRJNA1124986.

The 16S rRNA sequencing of all stool samples was examined to identify those containing ≥ 3% of their 16S rRNA reads aligned with the genus *Staphylococcus*. Samples showing >3% of reads attributed to *Staphylococcus* were plated on Mannitol Salt Agar (Hardy Diagnostics, Santa Maria, CA, USA) to differentiate *Staphylococcus aureus* from coagulase-negative staphylococci; red/pink colonies were streaked on BBL Trypticase Soy Agar with 5% Sheep Blood (BD Bioscience, Sparks, MD, USA) to isolate individual colonies. Matrix-assisted laser desorption/ionization time-of-flight mass spectrometry (MALDI-TOF) was conducted on the isolated colonies to verify bacterial identification at the species level to *S. epidermidis* (Bruker MALDI Biotyper). A total of 21 gastrointestinal colonizing isolates derived from unique AML patient stools were determined to be *S. epidermidis*. All AML-origin bloodstream infection and GI-colonizing *Staphylococcus epidermidis* isolates can be found under BioProject PRJNA1262472, and all accession numbers are located in Table S1.

### 2.2. Whole-Genome Sequencing and Analysis of Invasive and GI-Colonizing S. epidermidis Isolates

Bacterial bloodstream infection (BSI)-positive AML patients were identified through collaboration between leukemia specialists and the clinical microbiology team at MDACC for all three patient cohorts [26,28,29]. A total of 23 bloodstream isolates were collected from AML patients. DNA was extracted from individual bacterial isolates using the MasterPure Gram-positive DNA purification kit (Lucigen, Middleton, WI, USA) according to the manufacturer’s protocol. Whole-genome sequencing (WGS) of the colonization and infectious *S. epidermidis* isolates from the PA13 and PA15 cohorts was conducted using the NovaSeq S4 platform with a 150 bp paired-end read protocol, while the PA14 cohort was sequenced on NextSeq500 with a 150 bp paired-end read protocol. The assemblies were compiled using SPAdes and annotated using the RAST tool kit (RASTtk) pipeline on BV-BRC (https://www.bv-brc.org, accessed on 12 December 2024) [4,8].

### 2.3. Comprehensive Genome Analysis

Genomic data from 70 *S. epidermidis* isolates were downloaded from the NCBI database and included in this study. The inclusion criteria were that the isolates had to have been identified as coming from a human host, have a known body site/source of collection to determine if there were infectious or colonizing isolates, and contain 5 or fewer contigs (to increase confidence in variant calling/SNP level variation).

A comprehensive genomic analysis was conducted on infectious, colonizing, and NCBI-derived isolates using the Bacterial and Viral Bioinformatics Resource Center (BV-BRC) to retrieve genomic data and assess the presence of virulence factors and antimicrobial resistance genes. Genome assembly was carried out using SPAdes, and the reference sequence used was *S. epidermidis* ATCC 12228, a non-infectious strain. The Comprehensive Antibiotic Resistance Database (CARD) was utilized to identify antimicrobial resistance genes (ARGs), while the Virulence Factor Database (VFDB) facilitated the analysis of virulence factors. The results from these databases were visualized as heatmaps using the R package “*pheatmap*” (version 1.0.13). Variation analysis was performed on the BV-BRC interface using the BWA-mem aligner (version 0.7.17) and FreeBayes SNP caller (version 1.3.7) for paired infection and gastrointestinal colonization isolates from the same patient. Multi-locus sequence typing (MLST) was performed through the PubMLST (https://pubmlst.org/organisms/staphylococcus-epidermidis, accessed on 14 January 2025). GraphPad Prism 9 was used for statistical testing, such as Fisher’s exact test comparing ARG and virulence factors between cohort and infection groups, as well as for the creation of box plots.

### 2.4. Whole-Genome Comparison of Colonizing Versus Infection Isolates

Whole-genome annotation of all 114 isolates was first conducted using Prokka (version 1.14.5), a command-line software that generates bacterial sequence annotations in GFF3 format [32]. Three isolates were dropped from this analysis as they did not produce usable Prokka files. Genome assemblies were first annotated with RESTtk on BV-BRC for general characterization, then re-annotated with Prokka to produce standardized GFF3 files required for the downstream analyses in Roary and Scoary (version 1.6.16). These GFF3 files enabled pan-genome construction using Roary, a widely used, high-speed pipeline for bacterial comparative genomics [33,34]. Whole-genome comparisons were performed between 111 isolates, including 55 colonizing and 56 infecting isolates. The pan-genome analysis was performed using Roary v3.13.0 to characterize core and accessory genomes among the isolates (Table S2). Core genes were defined as those present in at least 90% of the genomes. The analysis produced a core genome alignment and a gene presence/absence matrix. The differential genes identified were gated first by *p*-value, with a cut-off of 0.01, then by odds ratio, needing to be over 2 or under 1. Following this, if any of the categories contained fewer than 3 samples, those results were also removed. The summary statistics for the pan-genome analysis can be found in Table S2. The resulting gene_presence_absence.csv file from Roary was combined with a trait file, specifying whether isolates were infectious or colonizing, and submitted to Scoary to identify associations between annotated genes and the provided traits. Following the identification of differentially represented genes (infection vs. colonization), functional analysis of protein structure and protein family classification was performed using Phyre2 and InterProScan (version 5.72-103.0) to confirm annotation by Scoary, as well as to determine the potential function of hypothetical proteins [35,36].

### 2.5. Phylogenetic Tree Construction, Tree Parsing, and Comparative Analyses

FASTA files from the 114 *Staphylococcus epidermidis* isolates, including AML-derived bloodstream and gastrointestinal (GI) colonization isolates, as well as publicly available reference strains from the NCBI database, were uploaded to the Bacterial and Viral Bioinformatics Resource Center (BV-BRC). The BV-BRC program utilizes PATRIC global protein families (PGFams), and selected ones were used to determine the phylogenetic placement of the genomes. The protein sequences from these families were aligned with MUSCLE, and the nucleotides for each of those sequences were subsequently mapped to the protein alignment. The joint set of amino acids and nucleotide alignments were concatenated into a data matrix, and RaxML was used to analyze this matrix and construct a rooted maximum likelihood Codon Tree using all default parameters except for the number of genes that provide the nucleotide and amino acid sequence, which was changed from 100 to 1000. Maximum allowed deletions were set at 0, as was the maximum number of allowed duplications. The reference utilized was *S. epidermidis* ATCC 12228. Branch lengths (substitutions per site) were inferred, and bootstrap support values were calculated to assess clade confidence. The tree was exported in both PhyloXML and Newick formats for analysis and visualization.

## 3. Results

### 3.1. Phylogenetic Analyses of Isolates

**Figure 1:**
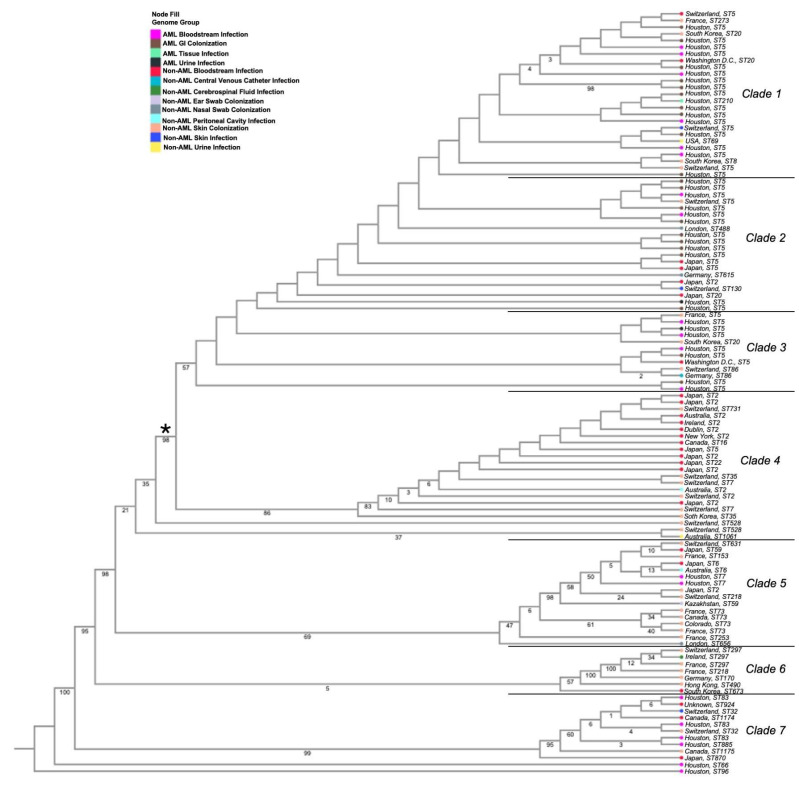
A phylogenetic tree of 114 *Staphylococcus epidermidis* isolates from AML and non-AML sources. A rooted maximum likelihood tree was constructed using the alignment of *S. epidermidis* isolates from AML patient cohorts and publicly available sequences. Branches are created with bootstrap support values to represent statistical confidence. Isolates are color-coded by isolate body site source and origin. AML-derived isolates include bloodstream infection (hot pink), AML tissue infection (mint), AML urine infection (black), and AML gastrointestinal colonization (brown). NCBI-derived isolates are grouped by bloodstream infection (red), central venous catheter infection (mid-toned blue), cerebrospinal fluid infection (dark green), ear swab colonization (pastel purple), nasal swab colonization (grey), peritoneal cavity infection (light blue), skin colonization (peach), skin infection (dark blue), and urinary infection (yellow). A major bifurcation appears around the 7th branch from the root (denoted by a *), where most AML-derived isolates cluster away from non-AML-derived isolates.

The whole-genome phylogenetic reconstruction of 114 *S. epidermidis* strains was built on gene and protein sequences for 1000 genes [37]. The whole-genome phylogeny tree shows seven clades (Figure 1, Table S1). Clade 1 is strongly composed of ST5 isolates, primarily from AML bloodstream and AML GI colonization isolates. There are, however, several non-AML bloodstream isolates in this clade also from the ST5 lineage that span various body sites and geographic locations. Clade 2 also consists almost exclusively of ST5 isolates and is mostly comprised of AML GI colonization isolates, non-AML bloodstream isolates, and a few AML bloodstream isolates. Clade 3 shows a diverse mix of isolates, featuring some non-AML skin colonizers, AML bloodstream infections, AML urine infections, non-AML bloodstream, and non-AML peritoneal cavity infectious isolates. This clade also features a majority of ST5 isolates.

Clades 4 through 7 feature primarily non-AML-derived isolates but also include a small number of AML bloodstream and AML GI strains. Clade 4 is made primarily of non-AML bloodstream infectious isolates and non-AML skin-colonizing isolates, with ST2 being the primary sequence type. Clade 5 is dominated by non-AML skin-colonizing isolates with a few entries from bloodstream, nasal, peritoneal, and AML bloodstream sources. Clade 6 is composed almost entirely of non-AML skin-colonizing isolates, with just one bloodstream and CSF isolate. Clade 7 clusters AML and non-AML bloodstream isolates alongside a few skin-colonizing strains, suggesting a more infection-focused lineage in this clade.

A major bifurcation appears around the seventh branch from the root (denoted by a *), where most AML-derived isolates cluster away from non-AML-derived isolates. ST5 and ST73 are the most common sequence types among non-AML colonization isolates (both 4/37), and ST5 is the only strain type representative of AML GI colonization isolates. While ST5 is also the most prevalent sequence type amongst AML infection isolates (14/23), ST2 is the most prevalent sequence type among non-AML infection isolates (11/33). When analyzing the phylogeny of MD Anderson AML only isolates by classification (infection or colonization) and by year, no obvious patterns emerged. Isolates seemed to cluster together primarily by classification, but the year of isolate collection did not appear to have an obvious impact on isolate phylogeny (Figure S1), suggesting that transmission within the inpatient setting was unlikely.

### 3.2. Differences in Antibiotic Resistance Genes and Virulence Factors Between Isolate Groups

**Figure 2:**
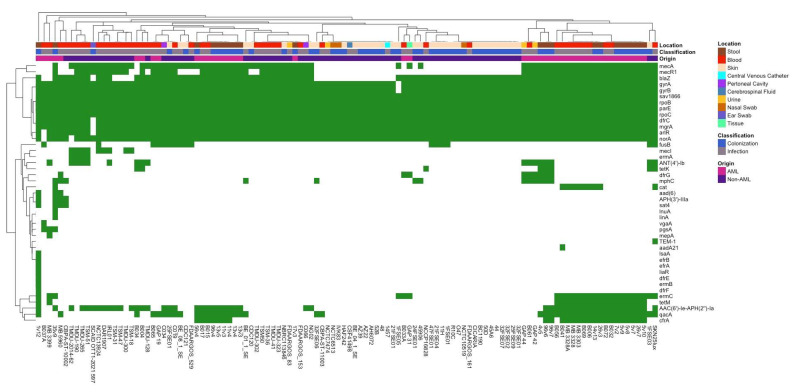
A heatmap depicting the distribution and clustering of antibiotic resistance genes (ARGs) across various isolates. ARG presence (green) and absence (white) are shown for individual isolates (columns) and genes (rows). The metadata above indicate isolate characteristics, including sample collection body site location, clinical classification, and isolate origin.

A comparative analysis of antimicrobial resistance genes (ARGs) and virulence factors across all isolates revealed significant differences in several key genes. To better visualize these patterns, binary heat maps were created to cluster the data, allowing for a clearer representation of gene prevalence across different sample origins and classifications (Figure 2). When examining observations between AML and non-AML isolates, infection-associated isolates, particularly those derived from blood and central venous catheters, grouped together and showed elevated levels of resistance genes, including *mecA*, *mecR1*, *qacA*, *erm A/B*, and *tetK*. Additional resistance genes such as *ermA*, *tetM*, *cfrA*, and *ANT(4′)-Ib* were also detected in subsets of infection isolates, reflecting a broader ARG repertoire. In contrast, skin-colonizing isolates clustered separately and often lacked *mecR1*, *qacA*, *ermC*, *tetM*, and *cfrA*. Non-AML isolates displayed variable patterns, with some skin and urinary isolates clustering with bloodstream infection isolates due to shared ARGs.

To better understand the connection between antibiotic resistance genes and infection status, we used Fisher’s exact test and the Chi-square test to determine if certain genes were significantly related to either an isolate’s origin (AML or non-AML) or classification (infection or colonization). In doing so, we observed several differences in the prevalence of different genes when compared by classification, suggesting that certain genes may be highly associated with infection status. Firstly, *mecI*, the regulator gene that represses *mecA*-mediated beta-lactam resistance, was found in 15.79% of infectious isolates but was completely absent from colonization isolates (*p* = 0.003) (Figure S2A). *mecA* (*p* = 0.003) and *mecR1* (*p* = 0.015) both also followed this pattern, being present in 77.19% and 64.91% of infectious isolates but only 50.88% and 42.11% of colonization isolates, respectively. Lastly, *ANT(4′)-lb*, an aminoglycoside resistance gene, was also enriched in infection isolates, reaching 22.80%, compared to only 7.02% prevalence among colonization isolates (*p* = 0.033) (Figure S2D).

A comparison of ARG presence between AML and non-AML isolates identified several genes with significant differences in prevalence. *cfrA*, a gene conferring resistance to linezolid, was detected in 56.82% of AML isolates but completely absent in non-AML isolates (*p* ≤ 0.0001) (Figure S3A). Similarly, *qacA*, an efflux pump conferring antiseptic resistance, was significantly more prevalent in AML isolates (77.27%) compared to non-AML isolates (12.86%) (*p* ≤ 0.0001) (Figure S3B). *tetM*, conferring protection against tetracycline, was also detected exclusively in AML isolates (43.18%) but was absent in non-AML isolates (*p* ≤ 0.0001) (Figure S3C). Other notable ARGs with significantly higher presence in AML isolates compared to non-AML isolates include *ermC*, a gene that encodes a methyltransferase conferring resistance to macrolide, lincosamide, and streptogramin antibiotics (50% vs. 4.29%, *p* ≤ 0.0001); *mecR1*, a gene that encodes a sensor-transducer protein triggering *mecA* expression (81.82% vs. 35.71%, *p* ≤ 0.0001); and *AAC(6′)-Ie-APH(2″)-Ia*, a bifunctional enzyme-encoding gene that confers high-level resistance to aminoglycosides (72.72% vs. 24.29%, *p* ≤ 0.0001) (Figure S3D–F). *mecA*, a key methicillin resistance gene, was also significantly enriched in AML isolates (86.36%) compared to non-AML isolates (50.00%) (*p* ≤ 0.0001) (Figure S3G). In contrast, *blaZ*, encoding a beta-lactamase that hydrolyzes penicillin-class antibiotics, was highly prevalent in both groups but significantly more common among AML isolates (95.45% vs. 71.43%, *p* = 0.0013) (Figure S3H). The *dfrG* gene, which confers resistance to trimethoprim by encoding a dihydrofolate reductase variant, was significantly more common in AML isolates (*p* = 0.0001), being found in 9 AML isolates, but completely absent from non-AML isolates (Figure S3I). A similar pattern was observed for *cat*, a chloramphenicol acetyltransferase gene responsible for chloramphenicol resistance, which was almost detected in 9 AML isolates but only 1 non-AML isolate (*p* = 0.0008) (Figure S3J). Lastly, *mecI*, a transcriptional repressor of *mecA*, was significantly more enriched in non-AML isolates (*p* = 0.012) and completely absent from AML isolates.

**Figure 3:**
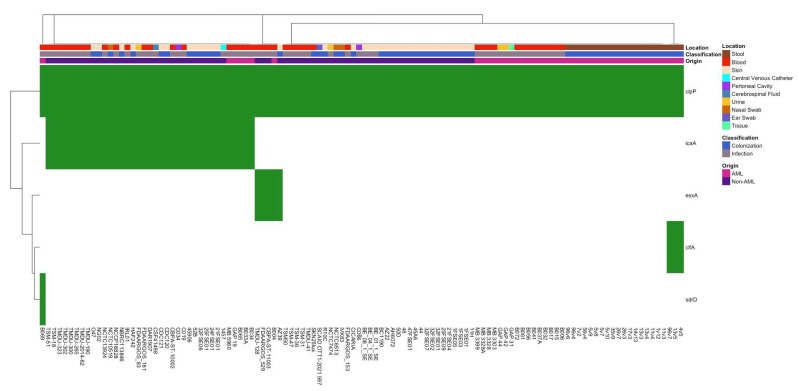
A heatmap illustrating the distribution and clustering of virulence factor genes identified in bacterial isolates. The presence (green) or absence (white) of virulence factors is indicated across individual isolates (columns) and genes (rows). The metadata above the heatmap describe the isolate characteristics, including sample collection body site locations, clinical classification, and isolate origins.

The presence of virulence genes was also analyzed according to classification (infection vs. colonization) and origin (AML vs. non-AML) (Figure 3). When comparing virulence gene presence across all AML and non-AML isolates (Figure 3), *icaA*, a gene associated with biofilm formation, was detected primarily in those classified as infections and non-AML isolates. *clfA*, a gene that encodes clumping factor A, which is involved in adherence to host tissues and biofilm formation, was observed only in AML colonization isolates, while *esxA* was present in both colonization and infection, as well as AML and non-AML isolates. Fisher’s exact test identified the *icaA* gene as significantly more abundant in both infection-derived isolates (*p* = 0.016) and non-AML origin isolates (*p* = 0.0001) (Figure S4A,B). These findings highlight the potential role of *icaA* in pathogenicity and suggest its enrichment in clinically relevant *S. epidermidis* isolates.

### 3.3. Whole-Genome Comparison of Infection vs. Colonization Isolates

A comparative analysis of gene presence across infectious and non-infectious *S. epidermidis* isolates identified 31 genes with statistically significant differences in prevalence among infectious vs. non-infectious isolates (*p* < 0.01, and OR < 0.3 or >3), suggesting potential roles in decreasing or increasing the odds of infection (Table 1). Several genes were significantly enriched in infectious isolates, including *xerC_2* (OR = 8.011, *p* = 0.004), *group_1639*, *group_4686-group_4688*, *group_9012*, and *group_9046* (all OR = 7.227, *p* = 0.008), many of which are annotated as hypothetical proteins, but are predicted to encode DNA-binding proteins, hydrolase inhibitors, or modulators of gene repression (Table 2). The IS6 family transposases *group_14970* (IS431mec) (OR = 5.284, *p* = 0.0009) and *mecA_2* (OR = 3.969, *p* = 0.0009) were also over-represented in infectious isolates, reaffirming the importance of methicillin resistance in pathogenic strains. Additional genes enriched among infection isolates included *group_2675* (signal peptidase IB) (OR = 3.767, *p* = 0.0011), *group_4522* (hydratase), and *ugpQ* (glycerophosphodiester phosphodiesterase) (both OR = 3.430, *p* = 0.0032).

Conversely, multiple genes were enriched in non-infectious isolates and associated with lower odds of developing infection, such as *group_2806* (OR = 0.016, *p* = 0.0002), *yhfS* (OR = 0.210, *p* = 0.0005), *group_1377* (OR = 0.250, *p* = 0.00405), *sps_B* (OR = 0.0249, *p* = 0.0006), *group_2646* (OR = 0.148, *p* = 0.0081), *group_3670* (OR = 0.148, *p* = 0.0081), *group_697* (OR = 0.293, *p* = 0.0046), *group_5697* (OR = 0.108, *p* = 0.001), and *group_1486* (OR = 0.210, *p* = 0.0017), all of which are implicated in either recombination, actyl-CoA metabolism, or hydrolysis, which are pathways potentially relevant to metabolic adaptation. These included predicted lipases, oxidoreductases, and hydrolases, possibly reflecting functions more aligned with commensal behavior (Table 1 and Table 2).

## 4. Discussion

In this study, we aimed to determine whether *S. epidermidis* isolates from AML patients showed genomic differences from non-AML obtained isolates and whether infection-associated isolates demonstrated distinct genetic differences compared to colonizing strains. Through phylogenetic reconstruction, resistome and virulence profiling, and pan-genome analyses, we investigated 114 isolates representing AML and non-AML isolates, stemming from a variety of infection and colonization sites. By comparing these isolates, we aimed to identify the specific genomic traits, including antibiotic resistance determinants, virulence factors, and accessory gene content, which may play a role in facilitating the transition from harmless colonizer to opportunistic pathogen in immunocompromised patients.

While we did not see any noticeable patterns related to isolate collection years among AML isolates, sequence types (STs) generally clustered well within the phylogenetic tree, though not always as single monophyletic groups. Notably, ST5 and ST2, both of which are globally dominant and frequently associated with bloodstream infections, were distributed across multiple distinct but closely related subclusters rather than forming unified clades. This pattern likely reflects microevolutionary divergence or clonal expansion events within hospital settings. Several ST5 subclusters included isolates from geographically diverse locations, such as Houston (AML Isolates), France, and Switzerland. Similarly, ST2 isolates were found in several clades, suggesting diversification within this clinically relevant group. Other sequence types, such as ST73, ST30, and ST59, showed tighter, more cohesive clusters, often forming clear, well-supported monophyletic branches. These observations indicate that while ST designation correlates well with phylogenetic structure, within-ST diversity, especially among epidemic clones like ST5 and ST2, should not be overlooked.

A comparison of isolates from AML versus non-AML origins revealed notable genetic and functional distinctions. Phylogenetic analysis showed that AML isolates, regardless of infection status, tended to cluster more closely together than non-AML isolates. Non-AML isolates, in contrast, showed more diversity, appearing throughout multiple phylogenetic clades but demonstrating some clustering based on the classification of isolates. AML isolates also exhibited significantly higher prevalence of numerous resistance determinants, including *mecA*, *mecR1*, *cfrA*, *tetM*, *ermC*, *qacA*, and *AAC(6′)-le-APH(2″)-la* and several others. These findings support the idea that antimicrobial pressure is a major driver of resistance gene enrichment and that the treatment environment selects for multidrug-resistant subpopulations [38]. Notably, *cfrA*—a key last-resort antibiotic frequently used to treat methicillin-resistant *S. epidermidis* infections—was completely absent from non-AML isolates but found in 56.82% of AML-derived strains, suggesting not only strong selective pressure within this immunocompromised cohort but also the circulation of hospital-adapted lineages where linezolid resistance has become increasingly reported [39]. Similarly, *tetM*, a ribosomal protection protein that mediates tetracycline resistance, was detected only in AML isolates. While tetracyclines are not often utilized as a primary treatment for bloodstream infections, their broad-spectrum use in empiric regimens may create an environment allowing for co-selection, likely through mobile genetic elements, and the continued presence of this resistance element in hospital-associated strains [38]. These findings suggest that a more uniformly resistant *S. epidermidis* population has proliferated in the hospitalized/treatment AML patient population, capable of thriving under prophylactic and empirical treatment regimens. These observations align with recent studies that found high antimicrobial resistance gene carriage among *S. epidermidis* isolates in hematologic malignancy patients and ICU cohorts [40,41,42]. However, the degree of ARG enrichment in AML isolates observed here, especially for *cfrA* and *tetM*, suggests that selective pressure in AML patients may be even more intense than previously appreciated. Although this could be an artifact of all AML isolates being collected from one institution and, thus, represent clonal transmission, it could also suggest that AML-colonizing isolates have adapted to the selective pressures caused by intensive chemotherapy and antibiotic exposure, given that all AML GI colonizers, some non-AML colonizers, and the majority of AML-infectious isolates are derived from the same lineage (ST5) but differ in their genomic content.

Analyses comparing different isolate classifications (infectious or colonizing) identified distinct features that could correlate with pathogenic potential. Infection-derived isolates showed significant enrichment for multiple ARGs—specifically, *mecI* (15.79% in infectious isolates compared to 0% in colonizing isolates), *mecA* (77.19% vs. 50.88%), *mecR1* (64.91% vs. 42.11%), and *ANT(4′)-lb* (22.80% vs. 7.02%). The increased enrichment of these specific genes in infectious isolates highlights the role that resistance to many commonly used antibiotics plays in distinguishing infectious strains from colonizing strains. The *mecA* gene, encoding PBP2a, and its regulator *mecR1* confer resistance to nearly all beta-lactam antibiotics, which are often used as first-line empirical therapies for suspected bloodstream infections [7,12,24]. The increased presence of these genes in infection-derived isolates suggests that these isolates are better equipped to survive and proliferate under empirical antibiotic treatment for other etiological agents of infection. The enrichment of these resistance genes in infection-derived isolates also contributes to the belief that increased antibiotic tolerance may be a key factor in allowing *S. epidermidis* to transition from its role as a benign colonizer to an opportunistic pathogen, particularly in the context of bloodstream or device-associated infections.

Pan-genome analysis identified 12 genes associated with significantly higher odds of infection. These included *xerC_2*, *group_1639*, *group_4684-group_4688*, *group_9012*, *and group_9046*, which had predicted functions related to recombination, transposases, DNA binding, transcriptional repression, and stress response, suggesting that horizontal gene transfer and genomic plasticity may contribute to potential infection [24,25,43]. *mecA_2* also showed significantly higher odds in infectious isolates, demonstrating how prevalent antibiotic resistance traits are in these strains. When examining non-infectious isolates, several genes, including *group_2806*, *yhfS*, *group_1377*, *sps_B*, *group_2646*, *group_3670*, *group_697*, *group_5697*, and *group_1486*, were associated with reduced odds of infections. These genes were predicted to encode lipases, oxidoreductases, and hydrolases, all of which are consistent with non-pathogenic, commensal function. These contrasting gene patterns between infectious and colonizing isolates reinforce the idea that adaptation to stress and resistance, rather than traditional virulence, underpins the clinical emergence of *S. epidermidis*. These predicted genes represent high-priority candidates for future experimental validation to better understand their role in the pathogenesis of *S. epidermidis*. Our results contrast with earlier findings suggesting that the roles of biofilm-associated and adhesion-related genes were highly important for driving virulence in *S. epidermidis* pathogenesis [44,45]. While our cohort of patients did identify *icaA* as being enriched in infectious isolates, many other virulence genes appeared infrequently among isolates and were not strongly predictive of infection, reinforcing the emerging view that resistance and metabolic adaptability play a more central role in the pathogenic transition of *S. epidermidis*. Finally, many significantly associated genes remained uncharacterized or were annotated as hypothetical proteins with either no matches or low-confidence predictions.

To further investigate the potential genetic relationship between colonizing and infecting isolates within the same patient, SNP-level variation was performed between five colonization isolates and one infectious isolate from patient 5 and one colonization isolate and three infectious isolates from patient 17. In one AML patient with paired gastrointestinal and bloodstream isolates, the isolates belonged to different sequence types entirely, indicating independent origins. In the second patient, both colonizing and infectious isolates were classified as ST5; however, the colonizing isolate differed by approximately 1200 SNPs from each of the three bloodstream isolates, which were highly genetically similar to one another (<100 SNPs apart). This very limited analysis further highlights that infection status is not determined solely by lineage, but rather by the accumulation of adaptive genomic features. Moreover, these results also potentially challenge the assumption that all *S. epidermidis* bloodstream infections in immunocompromised patients generally arise from the translocation of a dominant gastrointestinal, mucosal, or skin colonizer [46,47]. Longitudinal strain tracking of multiple biogeographic sites over time would need to be performed to determine the true origin of *S. epidermidis* systemic infections in immunocompromised mucosally damaged individuals.

This study has several limitations. First, although our dataset included 114 *S. epidermidis* isolates, only two AML patients had paired gastrointestinal and bloodstream isolates available for SNP-level comparison, limiting our ability to generalize within-host evolutionary conclusions. Second, all AML patient isolates were obtained from a single institution, potentially restricting the geographic and clinical diversity of the AML-associated strains captured in this study. Due to the fact that all AML isolates came from only one institution, multicenter studies would be needed to generalize the comparisons between AML colonization and infection isolates and AML and non-AML isolates. Additionally, the functional analysis on the proteins identified from the comparative genomic analysis yielded uncharacterized or “hypothetical” proteins, which we annotated further. However, without further functional validation, we are limited in our ability to completely understand the role these genes play in the pathogenesis of *S. epidermidis* infection.

Together, these data suggest that pathogenicity in *S. epidermidis* likely reflects both between-host transmission of resistant, hospital-adapted strains and within-host diversification of commensal lineages, particularly under antimicrobial and immunological pressure [4,8,20,21]. These findings also suggest that the pathogenicity of *S. epidermidis* is not driven by classical virulence factors but by genomic traits that enable survival, immune evasion, and persistence under intense environmental conditions. Understanding these features is critical for improving infection risk assessment, refining antibiotic stewardship strategies, and developing novel interventions to mitigate infection in vulnerable immunocompromised populations.

## Figures and Tables

**Table 1:** The association of genes with infectious versus non-infectious isolates identified through Scoary.

Gene	Annotation	Infectious (Present)	Non-Infectious (Present)	Infectious (Not Present)	Non-Infectious (Not Present)	Odds Ratio	*p*-Value
*xerC_2*	Tyrosine recombinase XerC	13	2	43	53	8.012	0.00414
*group_1639*	Hypothetical protein	12	2	44	53	7.227	0.00809
*group_4686*	Hypothetical protein	12	2	44	53	7.227	0.00809
*group_4687*	Hypothetical protein	12	2	44	53	7.227	0.00809
*group_4688*	Hypothetical protein	12	2	44	53	7.227	0.00809
*group_9012*	Hypothetical protein	12	2	44	53	7.227	0.00809
*group_9046*	Hypothetical protein	12	2	44	53	7.227	0.00809
*group_14970*	IS6 family transposase IS431mec	22	6	34	49	5.284	0.00085
*mecA_2*	PBP2a family beta-lactam-resistant peptidoglycan transpeptidase MecA	43	25	13	30	3.969	0.00090
*group_2675*	Signal peptidase IB	34	16	22	39	3.767	0.00113
*group_4522*	Hypothetical protein	43	27	13	28	3.430	0.00318
*ugpQ*	Glycerophosphodiester phosphodiesterase, cytoplasmic	43	27	13	28	3.430	0.00318
*group_697*	2-succinylbenzoate--CoA ligase	11	25	45	30	0.293	0.00458
*group_2478*	Hypothetical protein	7	19	49	36	0.271	0.00731
*arsB_2*	Arsenical pump membrane protein	28	44	28	11	0.250	0.00135
*group_1377*	Hypothetical protein	7	20	49	35	0.250	0.00405
*spsB_3*	Signal peptidase IB	20	38	36	17	0.249	0.00059
*yhfS*	Putative acetyl-CoA C-acetyltransferase YhfS	29	46	27	9	0.210	0.00049
*group_1486*	Putative protein YxeI	6	20	50	35	0.210	0.00165
*group_5702*	Hypothetical protein	5	18	51	37	0.202	0.00227
*group_15014*	IS200/IS605 family transposase ISSep3	3	13	53	42	0.183	0.00701
*group_2646*	Ribulose-5-phosphate reductase 2	2	11	54	44	0.148	0.00809
*group_3670*	DNA-invertase hin	2	11	54	44	0.148	0.00809
*group_3678*	Putative protein	2	11	54	44	0.148	0.00809
*group_5714*	Hypothetical protein	2	11	54	44	0.148	0.00809
*group_5814*	Hypothetical protein	2	11	54	44	0.148	0.00809
*group_3608*	Hypothetical protein	2	13	54	42	0.120	0.00208
*group_5695*	Hypothetical protein	2	13	54	42	0.120	0.00208
*group_2806*	Hypothetical protein	3	18	53	37	0.116	0.00022
*group_5693*	Hypothetical protein	2	14	54	41	0.108	0.00102
*group_5697*	Hypothetical protein	2	14	54	41	0.108	0.00102

Gene names are italicized.

**Table 2:** Functional analyses and annotations of candidate genes identified by SCOARY analyses as differentially abundant in infectious isolates and non-infectious isolates.

Gene	SCOARY Annotation	Phyre Annotation (Confidence)	InterproScan Annotation
*xerC_2*	Tyrosine recombinase XerC	Tyrosine recombinase xerA (100)	Phage Integrase
*group_1639*	Hypothetical Protein	DNA Binding Protein (100)	No Prediction
*group_4686*	Hypothetical Protein	Hydrolase Inhibitor (52.9)	No Prediction
*group_4687*	Hypothetical Protein	Repression Modulator (99.7)	Unknown Function
*group_4688*	Hypothetical Protein	Arsenic Responsive Repressor (78.2)	No Prediction
*group_9012*	Hypothetical Protein	Viral Protein (98.9)	No Prediction
*group_9046*	Hypothetical Protein	Structural Protein (97.0)	No Prediction
*group_14970*	IS6 family transposase IS431mec	Mutator family transposase (98.4)	IS6 Family Transposase
*mecA_2*	PBP2a family beta-lactam-resistant peptidoglycan transpeptidase MecA	Penicillin-binding protein 2A (100)	Beta-Lactamase
*group_2675*	Signal peptidase IB	Hydrolase (100)	Signal Peptidase
*group_4522*	Hypothetical Protein	Thioesterase/thiol ester dehydrase-isomerase (100)	Hydratase/Beta-methylmalyl-CoA Dehydratase
*ugpQ*	Glycerophosphodiester phosphodiesterase, cytoplasmic	Glycerophosphodiester phosphodiesterase (100)	No Prediction
*group_697*	2-succinylbenzoate--CoA ligase	Ligase (100)	No Prediction
*group_2478*	Hypothetical Protein	RAS and A-Factor Converting Enzyme (99.8)	No Prediction
*arsB_2*	Arsenical pump membrane protein	Transport Protein (99.7)	Arsenical pump membrane protein
*group_1377*	Hypothetical Protein	Hydrolase (100)	Exonuclease
*spsB_3*	Signal peptidase IB	Hydrolase (100)	Signal Peptidase
*yhfS*	Putative acetyl-CoA C-acetyltransferase YhfS	Beta-ketothiolase (100)	Thiolase-like superfamily
*group_1486*	Putative protein YxeI	Hydrolase (100)	Peptidase C59 Family Enzyme
*group_5702*	Hypothetical Protein	Lyase (14.4)	No Prediction
*group_15014*	IS200/IS605 family transposase ISSep3	ISHP608 Transposase (100)	No Prediction
*group_2646*	Ribulose-5-phosphate reductase 2	Oxidoreductase (100)	No Prediction
*group_3670*	DNA-invertase hin	Hydrolase (100)	Site-specific recombinase resolvase
*group_3678*	Putative protein	Unknown Protein (100)	CSA family
*group_5714*	Hypothetical Protein	Putative regulator of transfer genes (99.8)	No Prediction
*group_5814*	Hypothetical Protein	Myeloproxidase inhibitor SPIN (66.6)	No Prediction
*group_3608*	Hypothetical Protein	Signaling Protein (78.4)	No Prediction
*group_5695*	Hypothetical Protein	Prohormone (59.2)	No Prediction
*group_2806*	Hypothetical Protein	Tumor necrosis factor ligand superfamily member	No Prediction
*group_5693*	Hypothetical Protein	Viral Protein (8.7)	No Prediction
*group_5697*	Hypothetical Protein	Acetyltransferase (98.3)	No Prediction

Gene names are italicized.

## Data Availability

All data generated for this study can be found in the NCBI sequence read archive. Specifically, all AML-origin infectious and colonizing *Staphylococcus epidermidis* isolate sequences generated as part of this study can be found under BioProject PRJNA1262472. All isolate sequences not generated by the authors are readily available on NCBI. All accession numbers of the *S. epidermidis* strains analyzed herein are shown in Table S1. The 16S rRNA sequences used to determine which stools should be cultured for *Staphylococcus epidermidis* gastrointestinal colonizing isolates are deposited under the BioProjects PRJNA352060, PRJNA526551 (16S rRNA sequencing PA13-0339), and PRJNA1124986 (16S rRNA sequencing PA15-0780) as part of previously published studies. Code for this project can be found at https://github.com/stephmcm/Staph-epi-Paper, repository created on 19 February 2025.

## Supplementary Materials

The following supporting information can be downloaded at https://www.mdpi.com/article/10.3390/pathogens14070627/s1, Table S1: Isolates used in analyses; Table S2: Summary Statistics from Roary Analysis; Figure S1: Phylogenetic analysis of AML-only isolates by classification and by year. Figure S2: Distribution of significant antimicrobial resistance genes in *S. epidermidis* isolates, classified by infection or colonization via Fisher’s exact test and the Chi-square test; Figure S3: Comparison of significant antimicrobial resistance genes between AML isolates and NCBI-sourced isolates via Fisher’s exact test; Figure S4: Prevalence of biofilm-associated virulence gene *icaA* across isolate classification and origin.
