# Supplementary material for: Staphylococcus epidermidis in Acute Myeloid Leukemia: A Comparative Genomic Study Against Non-AML Isolates
Source: Pathogens. 2025 Jun 24;14(7):627. doi: 10.3390/pathogens14070627 (PMC12301045; doi:10.3390/pathogens14070627)
Supplement: Supplementary file 1 [file pathogens-14-00627-s001.zip › pathogens-3708023-supplementary.pdf]

Table S1: Isolates used in analyses.

Sample ID	Origin	Classification	Site	Location	Sequence Type (ST)	Accession Numbers
**1v12**	AML	Colonization	Stool	Houston, TX	5	SAMN48455663
**11v3**	AML	Colonization	Stool	Houston, TX	5	SAMN48455664
**11v4**	AML	Colonization	Stool	Houston, TX	5	SAMN48455665
**13v4**	AML	Colonization	Stool	Houston, TX	5	SAMN48455666
**13v3**	AML	Colonization	Stool	Houston, TX	5	SAMN48455667
**13v5**	AML	Colonization	Stool	Houston, TX	5	SAMN48455668
**14v13**	AML	Colonization	Stool	Houston, TX	5	SAMN48455669
**17v3**	AML	Colonization	Stool	Houston, TX	5	SAMN48455670
**26v3**	AML	Colonization	Stool	Houston, TX	5	SAMN48455671
**26v7**	AML	Colonization	Stool	Houston, TX	5	SAMN48455672
**35v9**	AML	Colonization	Stool	Houston, TX	5	SAMN48455673
**4v5**	AML	Colonization	Stool	Houston, TX	5	SAMN48455674
**5v10**	AML	Colonization	Stool	Houston, TX	5	SAMN48455675
**5v7**	AML	Colonization	Stool	Houston, TX	5	SAMN48455676
**5v8**	AML	Colonization	Stool	Houston, TX	5	SAMN48455677
**5v9**	AML	Colonization	Stool	Houston, TX	5	SAMN48455678
**56v4**	AML	Colonization	Stool	Houston, TX	5	SAMN48455679
**7v2**	AML	Colonization	Stool	Houston, TX	5	SAMN48455680
**96v4**	AML	Colonization	Stool	Houston, TX	5	SAMN48455681
**96v6**	AML	Colonization	Stool	Houston, TX	5	SAMN48455682
**96v7**	AML	Colonization	Stool	Houston, TX	5	SAMN48455683
**B004**	AML	Infection	Blood	Houston, TX	885	SAMN48455684
**B006**	AML	Infection	Blood	Houston, TX	5	SAMN48455685
**B015**	AML	Infection	Blood	Houston, TX	5	SAMN48455686
**B017**	AML	Infection	Blood	Houston, TX	5	SAMN48455687
**B032**	AML	Infection	Blood	Houston, TX	5	SAMN48455688
**B033A**	AML	Infection	Blood	Houston, TX	7	SAMN48455689
**B034**	AML	Infection	Blood	Houston, TX	83	SAMN48455690
**B037A**	AML	Infection	Blood	Houston, TX	96	SAMN48455691
**B041**	AML	Infection	Blood	Houston, TX	5	SAMN48455692
**B056**	AML	Infection	Blood	Houston, TX	5	SAMN48455693
**B061**	AML	Infection	Blood	Houston, TX	5	SAMN48455694
**B065**	AML	Infection	Blood	Houston, TX	83	SAMN48455695
**B069**	AML	Infection	Blood	Houston, TX	5	SAMN48455696
**B072**	AML	Infection	Blood	Houston, TX	5	SAMN48455697

**GAP 19**	AML	Infection	Blood	Houston, TX	83	SAMN48455698
**MB 3303**	AML	Infection	Blood	Houston, TX	5	SAMN48455699
**MB 3328**	AML	Infection	Blood	Houston, TX	5	SAMN48455700
**MB 3328A**	AML	Infection	Blood	Houston, TX	5	SAMN48455701
**MB 3399**	AML	Infection	Blood	Houston, TX	66	SAMN48455702
**MB 5960**	AML	Infection	Blood	Houston, TX	7	SAMN48455703
**GAP 31**	AML	Infection	Tissue	Houston, TX	210	SAMN48455704
**GAP 42**	AML	Infection	Urine	Houston, TX	5	SAMN48455705
**GAP 44**	AML	Infection	Urine	Houston, TX	5	SAMN48455706
**SKN25lux**	Non-AML	Colonization	Blood	Bern, Switzerland	5	SAMN27305141
**SCAID OTT1-2021 597**	Non-AML	Colonization	Ear Swab	Almaty, Kazakhstan	59	SAMN20982454
**IVK83**	Non-AML	Colonization	Nasal Swab	Germany	615	SAMN23401985
**NCTC10519**	Non-AML	Colonization	Nasal Swab	London, United Kingdom	488	SAMEA3871774
**NCTC6513**	Non-AML	Colonization	Nasal Swab	London, United Kingdom	656	SAMEA3512674
**11H**	Non-AML	Colonization	Skin	France	153	SAMN18740291
**1FSE01**	Non-AML	Colonization	Skin	Bern, Switzerland	7	SAMN33711655
**1FSE03**	Non-AML	Colonization	Skin	Bern, Switzerland	5	SAMN33711654
**1FSE05**	Non-AML	Colonization	Skin	Bern, Switzerland	5	SAMN33711656
**21FSE01**	Non-AML	Colonization	Skin	Bern, Switzerland	86	SAMN33711667
**21FSE04**	Non-AML	Colonization	Skin	Bern, Switzerland	7	SAMN33711668
**24FSE01**	Non-AML	Colonization	Skin	Bern, Switzerland	35	SAMN33711671
**25FSE01**	Non-AML	Colonization	Skin	Bern, Switzerland	731	SAMN33711676
**25FSE09**	Non-AML	Colonization	Skin	Bern, Switzerland	631	SAMN33711677
**32FSE01**	Non-AML	Colonization	Skin	Bern, Switzerland	528	SAMN33711682
**32FSE02**	Non-AML	Colonization	Skin	Bern, Switzerland	218	SAMN33711684
**32FSE06**	Non-AML	Colonization	Skin	Bern, Switzerland	297	SAMN33711685
**32FSE07**	Non-AML	Colonization	Skin	Bern, Switzerland	528	SAMN33711686
**44**	Non-AML	Colonization	Skin	France	218	SAMN18740294
**45A6**	Non-AML	Colonization	Skin	France	5	SAMN18740296
**47FSE01**	Non-AML	Colonization	Skin	Bern, Switzerland	32	SAMN33711692

**48**	Non-AML	Colonization	Skin	France	273	SAMN18740293
**50D**	Non-AML	Colonization	Skin	France	253	SAMN18740292
**52B**	Non-AML	Colonization	Skin	France	297	SAMN18740297
**6-Sep**	Non-AML	Colonization	Skin	Changwon, South Korea	35	SAMN29006470
**AH6072**	Non-AML	Colonization	Skin	Colorado	73	SAMN30996408
**AZ22**	Non-AML	Colonization	Skin	Canada	73	SAMN17983072
**AZ39**	Non-AML	Colonization	Skin	Canada	1175	SAMN17983073
**BC1190**	Non-AML	Colonization	Skin	France	73	SAMN18740299
**CDC120**	Non-AML	Colonization	Skin	Seoul, South Korea	20	SAMN10473910
**CDC121**	Non-AML	Colonization	Skin	Seoul, South Korea	20	SAMN10473924
**CICARIA**	Non-AML	Colonization	Skin	Seongnam-si, South Korea	8	SAMN26495463
**HAF242**	Non-AML	Colonization	Skin	Hamburg, Germany	170	SAMN24585883
**NBRC113846**	Non-AML	Colonization	Skin	Japan	2	SAMN21163584
**NG02**	Non-AML	Colonization	Skin	Hong Kong	490	SAMN35671194
**O47**	Non-AML	Colonization	Skin	Bern, Switzerland	2	SAMN11960487
**R10C**	Non-AML	Colonization	Skin	France	73	SAMN18740290
**C019**	Non-AML	Infection	Blood	Perth, Australia	2	SAMN28460870
**CBPA-ST-10002**	Non-AML	Infection	Blood	Canada	16	SAMN17983070
**CBPA-ST-11003**	Non-AML	Infection	Blood	Canada	1174	SAMN17983071
**DAR1907**	Non-AML	Infection	Blood	New York	2	SAMN07444319
**FDAARGOS_153**	Non-AML	Infection	Blood	Washington D.C.	5	SAMN03996299
**FDAARGOS_161**	Non-AML	Infection	Blood	Washington D.C.	20	SAMN03996306
**FDAARGOS_529**	Non-AML	Infection	Blood	Unknown	924	SAMN10163224
**IRL01**	Non-AML	Infection	Blood	Ireland	2	SAMN09103952
**NCCP16828**	Non-AML	Infection	Blood	Jinju, South Korea	673	SAMN12721499
**NCTC13924**	Non-AML	Infection	Blood	Dublin, United Kingdom	2	SAMEA104318201
**TMDU-128**	Non-AML	Infection	Blood	Japan	870	SAMN26515352

**TMDU-190**	Non-AML	Infection	Blood	Japan	2	SAMN26515377
**TMDU-2014-62**	Non-AML	Infection	Blood	Japan	2	SAMN26518685
**TMDU-265**	Non-AML	Infection	Blood	Japan	2	SAMN26517650
**TMDU-300**	Non-AML	Infection	Blood	Japan	22	SAMN26517675
**TMDU-302**	Non-AML	Infection	Blood	Japan	20	SAMN26517680
**TMDU-323**	Non-AML	Infection	Blood	Japan	2	SAMN26517682
**TMDU-41**	Non-AML	Infection	Blood	Japan	5	SAMN26514226
**TSM-18**	Non-AML	Infection	Blood	Japan	2	SAMN26519684
**TSM-31**	Non-AML	Infection	Blood	Japan	6	SAMN26520624
**TSM-36**	Non-AML	Infection	Blood	Japan	5	SAMN26520678
**TSM-47**	Non-AML	Infection	Blood	Japan	59	SAMN26520700
**TSM-51**	Non-AML	Infection	Blood	Japan	5	SAMN26520719
**TSM50**	Non-AML	Infection	Blood	Japan	2	SAMN26520718
**1457**	Non-AML	Infection	Central Venous Catheter	Germany	86	SAMN02640609
**CSF41498**	Non-AML	Infection	Cerebrospinal Fluid	Ireland	297	SAMN09491332
**C034**	Non-AML	Infection	Peritoneal Cavity	Perth, Australia	2	SAMN28460979
**C066**	Non-AML	Infection	Peritoneal Cavity	Perth, Australia	6	SAMN28461093
**BE_01_1_SE**	Non-AML	Infection	Skin	Bern, Switzerland	5	SAMN32674114
**BE_04_1_SE**	Non-AML	Infection	Skin	Bern, Switzerland	130	SAMN32674115
**BE_08_1_SE**	Non-AML	Infection	Skin	Bern, Switzerland	32	SAMN32674116
**FDAARGOS_83**	Non-AML	Infection	Urine	USA	69	SAMN02934537
**NCTC7474**	Non-AML	Infection	Urine	Sydney, Australia	1061	SAMEA3505361

Table S2: Summary Statistics from Roary Analysis.

90%		
**Core Genes**	99% <= strains <= 100%	93
**Soft Core Genes**	95% <= strains < 99%	1586
**Shell Genes**	15% <= strains < 95%	969
**Cloud Genes**	0% <= strains < 15%	12394
**Total Genes**	0% <= strains <= 100%	15042

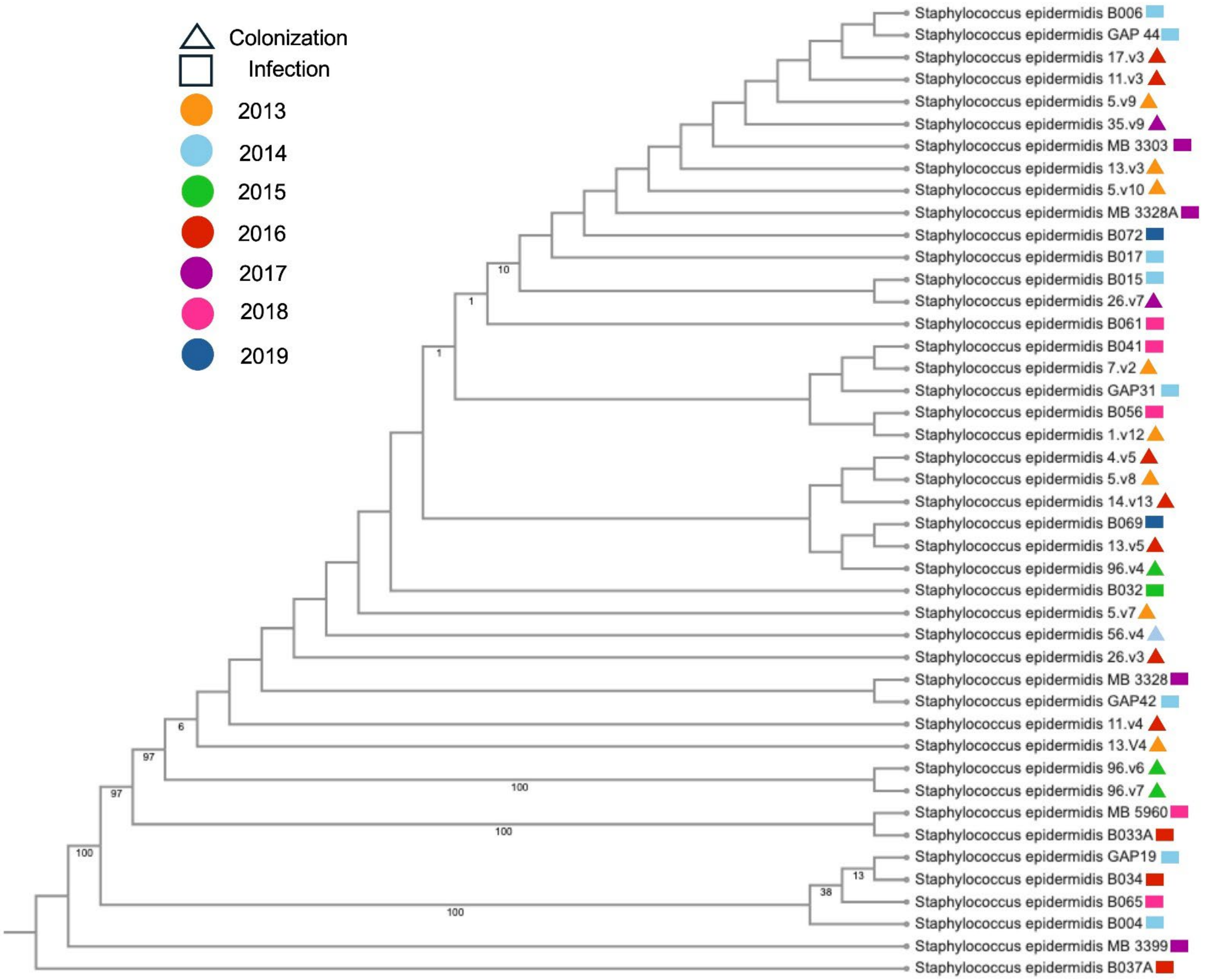

Figure S1: Phylogenetic analysis of AML only isolates by classification and by year.

Isolates are represented on the tree with either a triangle (colonization) or square (infection) and the year of collection is notated as orange (2013), light blue (2014), green (2015), red (2016), purple (2017), pink (2018), and dark blue (2019).

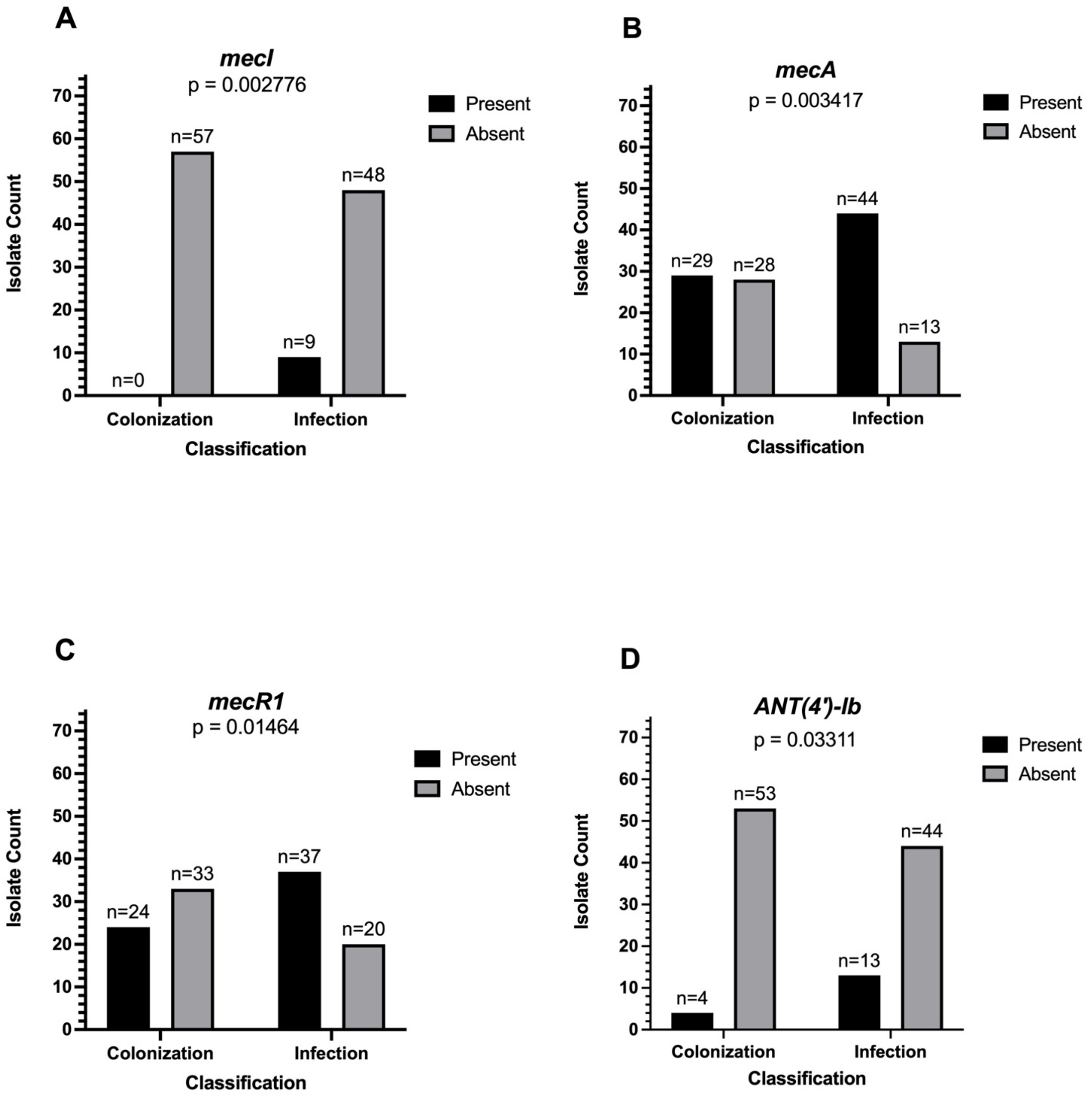

Figure S2: Distribution of significant antimicrobial resistance genes in *S. epidermidis* isolates, classified by infection or colonization via Fisher's exact test and Chi Square Test.

Bar graphs show the presence (black) or absence (grey) of resistance genes that are significantly different between infectious and colonizing isolate groups. The Y-axis represents the number of isolates, while the X-axis categorizes isolates as either colonizing or infectious. Significant genes include: (A) *mecI (p=0.0006837)*, (B) *mecA (p=0.005726),* (C) *mecR1 (p=0.01242)*, and (D) *ANT(4')-lb (p=0.03081).*

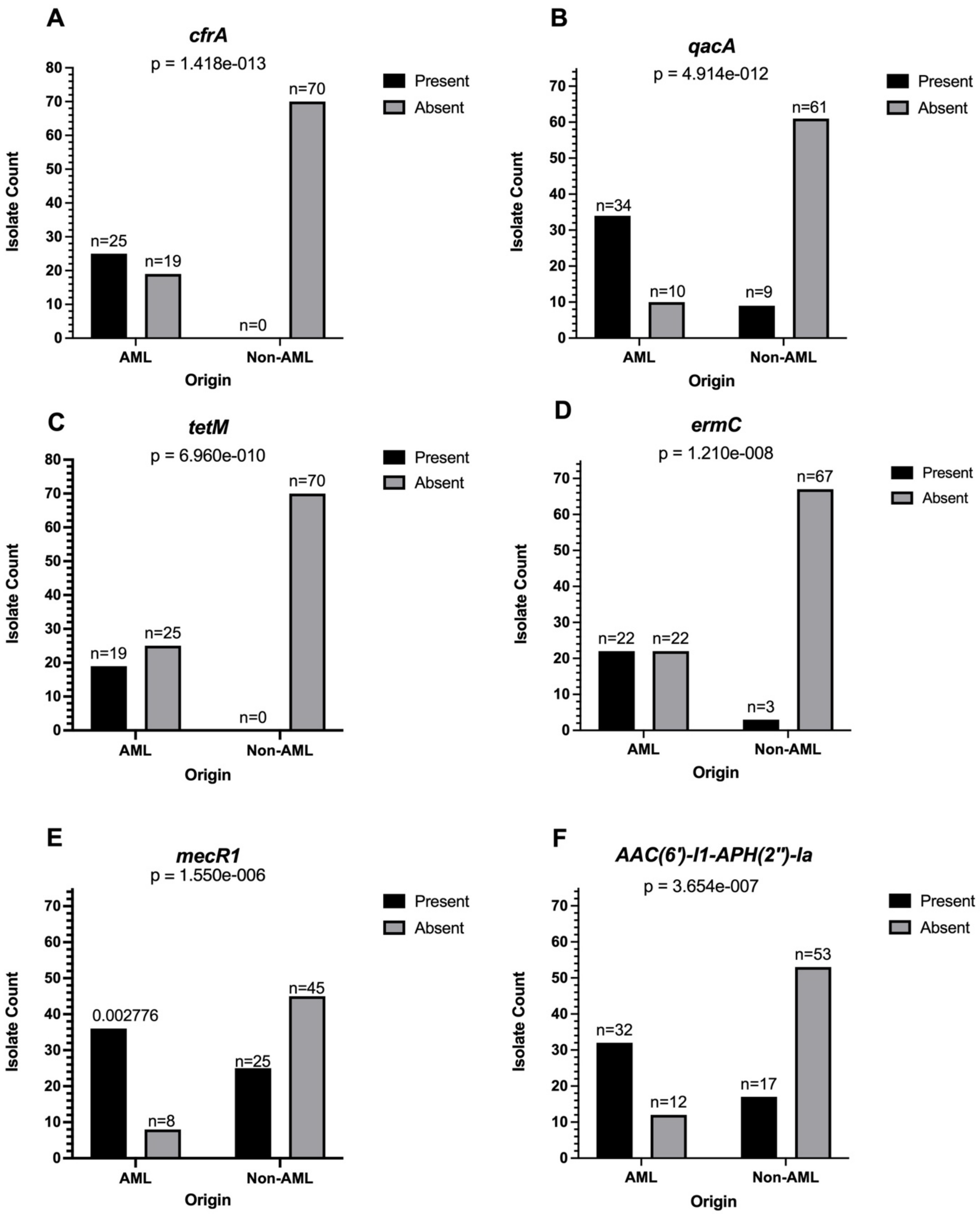
A
cfrA
p = 1.418e-013
Present
Absent
Isolate Count
n=25
n=19
n=0
n=70
AML
Non-AML
Origin
B
qacA
p = 4.914e-012
n=34
n=10
n=9
n=61
C
tetM
p = 6.960e-010
n=19
n=25
n=0
n=70
D
ermC
p = 1.210e-008
n=22
n=22
n=3
n=67
E
mecR1
p = 1.550e-006
0.002776
n=8
n=25
n=45
F
AAC(6')-I1-APH(2")-Ia
p = 3.654e-007
n=32
n=12
n=17
n=53

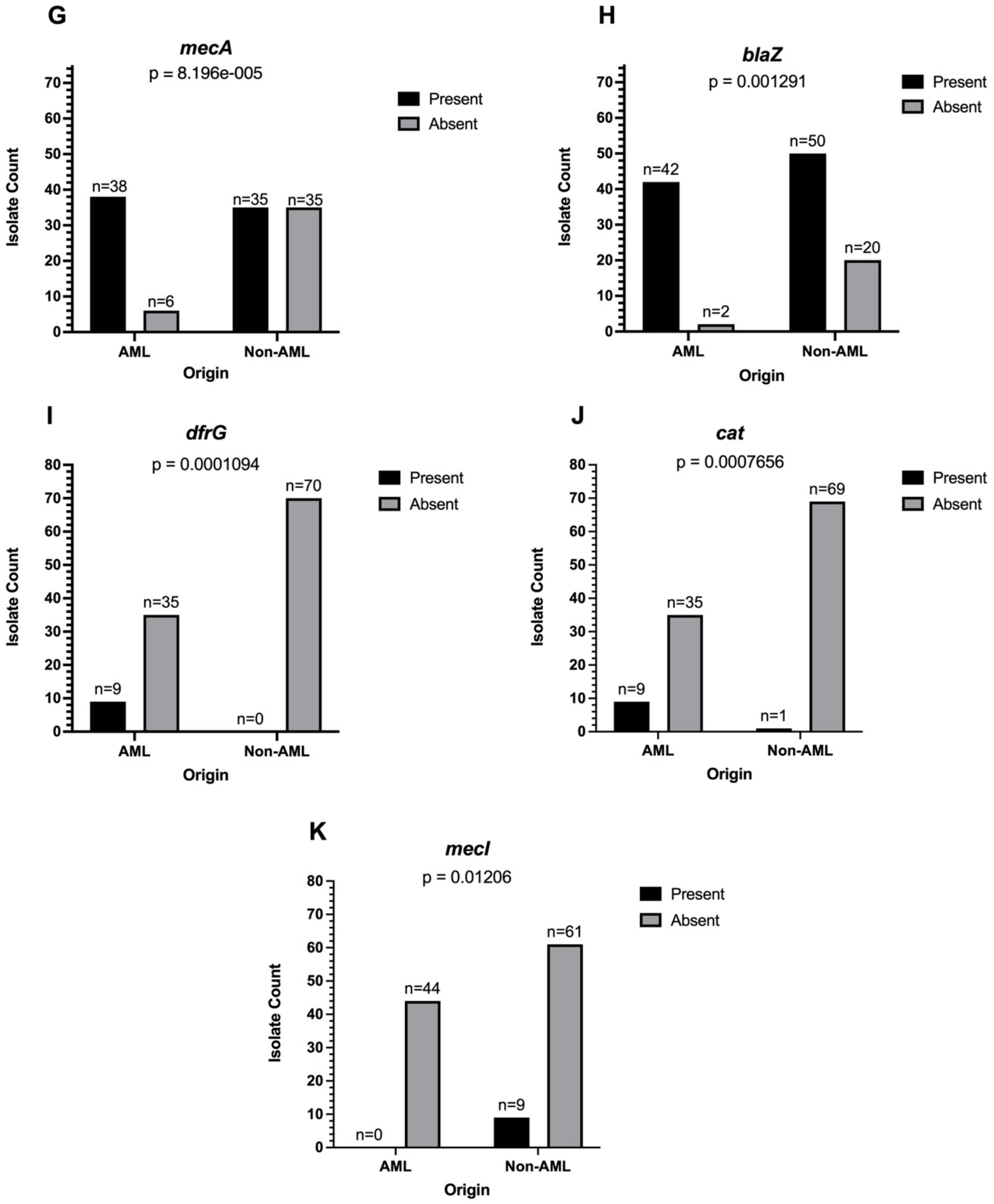

Figure S3: Comparison of significant antimicrobial resistance genes between AML isolates and NCBI-sourced isolates via Fisher's exact test.

Bar graphs illustrate the presence (black) and absence (grey) of significant resistance genes across isolates categorized by origin: AML isolates and publicly available isolates from non-AML patients (NCBI). The y-axis indicates the number of isolates. Significant genes include: (A) *cfrA (p=1.418e-13)*, (B) *qacA (p=4.914e-12)*, (C) *tetM (p=6.960e-10)*, (D) *ermC (p=1.210e-8)*, (E) *mecR1 (p=1.550e-6)*, (F) *AAC(6')-le-APH(2")-la (p=3.654e-7)*, (G) *mecA (p=8.196e-5),* (H) *blaZ (p=0.001291),* (I) *dfrG (p=0.0001094),* (J) *cat (p=0.0007656),* and (K) *mecI (p=0.01206).*

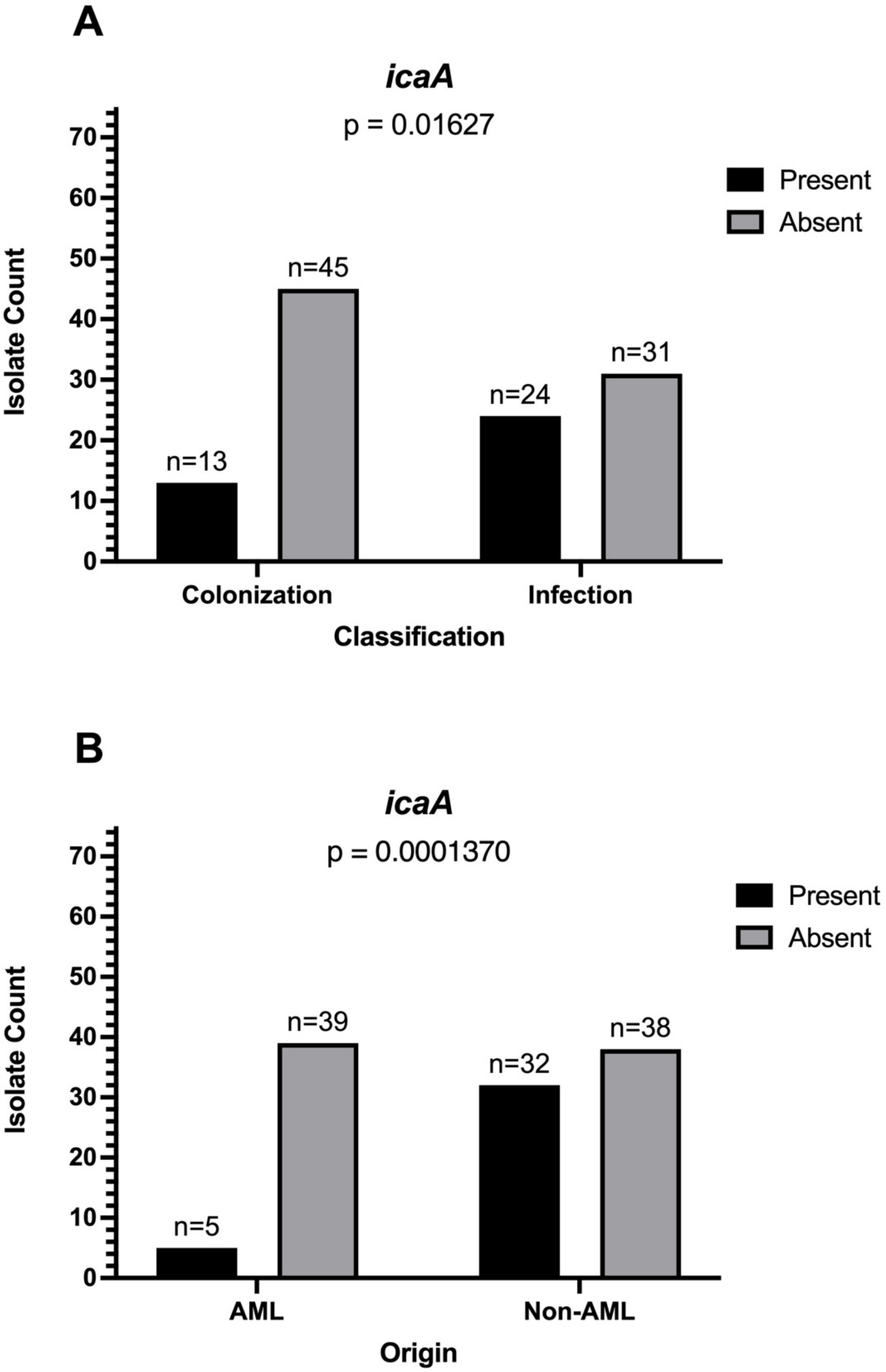

Figure S4: Prevalence of biofilm-associated virulence gene *icaA* across isolate classification and origins.

Bar graphs display the presence (black) and absence (grey) of the *icaA* gene in *S. epidermidis* isolates. (A) Comparison of *icaA* between colonization and infection isolates (*p=0.01627)*. (B) Comparison of *icaA* prevalence between AML isolates and NCBI non-AML isolates (*p=0.0001370).*
